# Bridging the Gap: A Quality Improvement Initiative to Enhance Support of Locally Employed Medical Registrars Through High-Fidelity Simulation

**DOI:** 10.7759/cureus.100130

**Published:** 2025-12-26

**Authors:** Sithamparanathan Skandan, Bipima Gurung, Khizar Hayat, Muhammad Iftikhar

**Affiliations:** 1 Medical Education and Simulation, Medway NHS Foundation Trust, Gillingham, GBR; 2 Medicine, Medway NHS Foundation Trust, Gillingham, GBR

**Keywords:** appraisal, clinical confidence, high-fidelity simulation, leadership development, locally employed doctor, medical registrar training, non-training grade, postgraduate medical education, registrar preparedness, speciality and specialist doctors

## Abstract

Introduction: Transition to the medical registrar role often lacks sufficient support, particularly for non-training grade doctors such as speciality and specialist (SAS) and locally employed doctors (LEDs). In the UK, while simulation-based education is well-established in early postgraduate stages for trainees in medicine, opportunities at the registrar transition level are limited for these groups. At Medway NHS Foundation Trust, no ongoing simulation existed for registrars outside formal training, despite the Royal College of Physicians' recommendations advocating equitable educational access for SAS and international medical graduate (IMG) doctors. This study describes a local needs assessment identifying training gaps and evaluates a targeted simulation course designed for SAS medical registrars with non-UK primary medical qualifications.

Methods: A three-step quality improvement (QI) approach was undertaken: (1) needs assessment via an anonymised online survey for SAS doctors, internal medicine training (IMT) stage 1 year 2 (IMT2) trainees, LEDs, and registrars, exploring demographics, clinical experience, self-perceived preparedness, and training gaps; (2) a bespoke one-day high-fidelity simulation course for six SAS medical registrars, targeting acute clinical scenarios and human factor skills, with live observation and facilitated debriefs focusing on clinical and non-technical competencies; (3) evaluation applying the Kirkpatrick model, including immediate post-course feedback assessing satisfaction and confidence change, and a one-month follow-up survey measuring behavioural changes, application of learning, and context barriers. Data were analysed quantitatively, and thematic analysis was applied to qualitative feedback.

Results: Baseline survey results showed 70.6% (n = 13) of respondents felt unprepared for registrar duties despite most having 1-3 years’ experience in the role, highlighting a significant gap in confidence. All valued simulation training, mentorship, and shadowing, with some SAS/LEDs considering stepping down due to role challenges. Post-simulation, all participants rated scenarios as realistic and relevant, all (n = 6) reported increased confidence in managing acute medical emergencies, and 83.3% (n = 5) described improvements in leadership and communication skills. One participant’s performance concern was identified, prompting targeted support. At one-month follow-up, respondents reported practical application of skills such as leadership, on-call and improved communication, alongside increased reflective practice. Limited peer feedback constrained a comprehensive behavioural change assessment. Conclusions: This QI project demonstrates the feasibility of using high-fidelity simulation to support medical registrars, particularly non-training grade doctors, by enhancing perceived confidence and identifying learning needs. Given the small sample size and reliance on self-reported outcomes, findings should be interpreted as preliminary and reflective of perceived educational value rather than objective competence. Nevertheless, simulation may represent a feasible and locally scalable educational approach to support equitable professional development for SAS and LEDs in line with the Royal College of Physicians' recommendations.

## Introduction

The medical registrar plays a pivotal role in acute hospital care, often acting as the clinical team leader responsible for managing medical emergencies, cardiac arrests, complex decision-making, and supervision of junior staff. This transition into higher responsibility is critical but frequently under-supported, particularly for doctors in non-training grade roles. While simulation-based education is well-established in early postgraduate training (e.g., internal medicine training (IMT), stage 1), structured simulation opportunities are often lacking at the point of transition to registrar level (IMT stage 2), especially for speciality and specialist doctors (SAS) and locally employed doctors (LEDs) [1-3].

Simulation has been shown to enhance both clinical competence and essential non-technical skills such as leadership, communication, and situational awareness [4,5]. At Medway NHS Foundation Trust, although IMT stage 1 year 2 (IMT2) trainees access a one-off ‘Ready for Registrar’ course, there is no ongoing simulation provision for registrars or equivalent doctors outside formal training pathways. This is despite the recent Royal College of Physicians' recommendations advocating equitable access to educational opportunities for LED and international medical graduate (IMG) doctors [3].

A local needs assessment was conducted to evaluate preparedness for the registrar role among trainees, SAS, and LEDs, and subsequently, a targeted simulation course for SAS medical registrars with non-UK primary medical qualifications (PMQ) was developed. This paper reports on the identified training gaps and evaluates the impact of the pilot simulation intervention.

## Materials and methods

A three-step quality improvement (QI) approach was undertaken, comprising the following: (1) a training needs assessment, (2) design and delivery of a simulation-based intervention, and (3) evaluation of its impact. Data were collected and analysed using Google® Forms.

Step 1: needs assessment

A cross-sectional online survey was conducted to assess the training needs and preparedness of doctors working as, or preparing to work as, medical registrars at Medway NHS Foundation Trust. The survey, created using Google Forms, was distributed via official departmental communication channels to all doctors in general medicine, including registrars, SAS, IMT2, and LED doctors, to capture a broad needs assessment. Participation was voluntary and anonymous.

The questionnaire comprised 14 questions, incorporating multiple choice, Likert scale, and open-ended questions, across five domains: demographics, preparedness, leadership confidence, debriefing access, and training needs. Items assessed participants’ level of training, duration of experience preparing for or working as a medical registrar, and perceived confidence in managing emergencies, leading teams, and making escalation decisions. Respondents were also asked about their previous exposure to shadowing, simulation, mentoring, and debriefing opportunities. Additional questions examined whether SAS doctors had ever considered stepping down to a senior house officer (SHO) role because of challenges encountered in the registrar post, and what specific areas of additional support or preparation they would find most beneficial. Questionnaire items were developed iteratively to align with the expected competencies and challenges of the medical registrar role. Draft items were reviewed by two medical registrars and a consultant physician to ensure relevance, clarity, and adequate coverage of key domains, thereby supporting content validity. Formal psychometric validation was not undertaken, as the tool was intended for formative evaluation within a QI framework.

Data analysis was descriptive. Likert-scale responses were summarised using medians and interquartile ranges, while categorical variables were reported as proportions and frequencies and free-text responses were reviewed for recurring themes. No inferential statistical testing was performed due to the exploratory nature of the study and small sample size. As a service evaluation and QI project, formal ethics approval was not required under Health Research Authority (HRA) guidance.

Step 2: intervention-simulation-based training

The intervention study population comprised SAS and LEDs working at the medical registrar level at a single district general hospital, who were invited to attend the simulation programme. No formal sample size calculation was undertaken, given the service-evaluation and QI design.

Informed by the needs assessment, a one-day high-fidelity simulation course was developed for six SAS medical registrars two months later, following approval by the local trust committee and confirmation of funding. The course was designed to reflect the clinical and non-technical demands of the acute medical registrar role. Each scenario was structured to incorporate both clinical and human factor challenges. All participants undertook the same high-fidelity simulation scenarios, delivered using a standardised structure including predefined learning objectives, scenario scripts, and a structured debriefing framework. Scenarios were facilitated by the same faculty group and followed an identical sequence to enhance reproducibility. The clinical component focused on managing deteriorating patients, making escalation decisions, and responding to common acute medical emergencies such as pneumothorax and arrhythmia. The human factors component emphasised leadership, prioritisation under pressure, difficult communication, conflict resolution, and multidisciplinary team coordination.

All simulation scenarios included pre-briefing guides, set scenario scripts, and key learning points to support reproducibility and future scalability of the programme. The materials were reviewed by senior medical registrars and a consultant physician to confirm clinical accuracy and content validity. The sessions were conducted within the hospital’s simulation department using a fully equipped simulated ward bay, featuring a high-fidelity adult patient simulator. Members of the simulation faculty acted in supporting roles, including nurse, patient voice, medical consultant, and switchboard operator, to replicate the complexity and realism of an acute medical environment.

Each participant led as the medical registrar in one high-fidelity acute medical simulation scenario, while other participants observed from a separate debriefing room. An acute medical consultant, two senior medical registrars, and members of the simulation faculty observed from the control room, which was separated from the simulated ward by a one-way mirror. Scenarios were live-streamed from the simulated ward to the debriefing room to facilitate real-time observation. Senior clinicians observed in a non-interventional role and were available for advice only if explicitly escalated to by the participant via a dedicated simulation telephone linking the simulated ward to the control room, mirroring real-world practice. Observers could not prompt, assist, or alter the clinical course, ensuring consistency of trainee experience while enabling focused observation for feedback. Participants were informed that live streaming was used solely to support observation and debriefing, and no recordings were retained.

Each simulation was followed by a structured debrief led by an experienced facilitator trained in dynamic debriefing methods. The debriefs focused on both human factors and trust-specific clinical pathways and protocols to ensure contextual relevance and address participant needs highlighted in the prior survey.

Step 3: evaluation of impact

Baseline questionnaires were completed immediately prior to the simulation course. Post-course evaluations were completed immediately following the session, with a follow-up questionnaire administered one month later to assess sustained perceived impact on confidence and clinical practice. Evaluation followed the Kirkpatrick model to assess both immediate and longer-term outcomes. Immediately after the course, participants completed an anonymous post-course evaluation form. This measured level 1 (reaction) outcomes, including satisfaction with the relevance, structure, facilitation, and environment of the course, and level 2 (learning) outcomes, through self-reported changes in confidence across domains such as leadership, escalation decisions, and clinical management. Free-text responses were reviewed thematically to identify perceived strengths and areas for improvement.

A one-month follow-up online survey was then distributed to all participants. This stage assessed behavioural change, corresponding to level 3 (behaviour) outcomes in the Kirkpatrick framework. Participants reflected on how they had applied the skills learned during the course in their clinical practice, particularly in leading teams, managing acutely unwell patients, and communicating effectively with colleagues. They were also given an online peer-feedback survey to distribute to colleagues who have worked with them over the past month, to seek feedback on whether there were observed changes in the participant’s practice. Qualitative data from open-ended responses were analysed thematically to identify examples of behavioural change, sustained confidence, and contextual barriers to implementation.

## Results

Step 1: needs assessment

**Figure 1:**
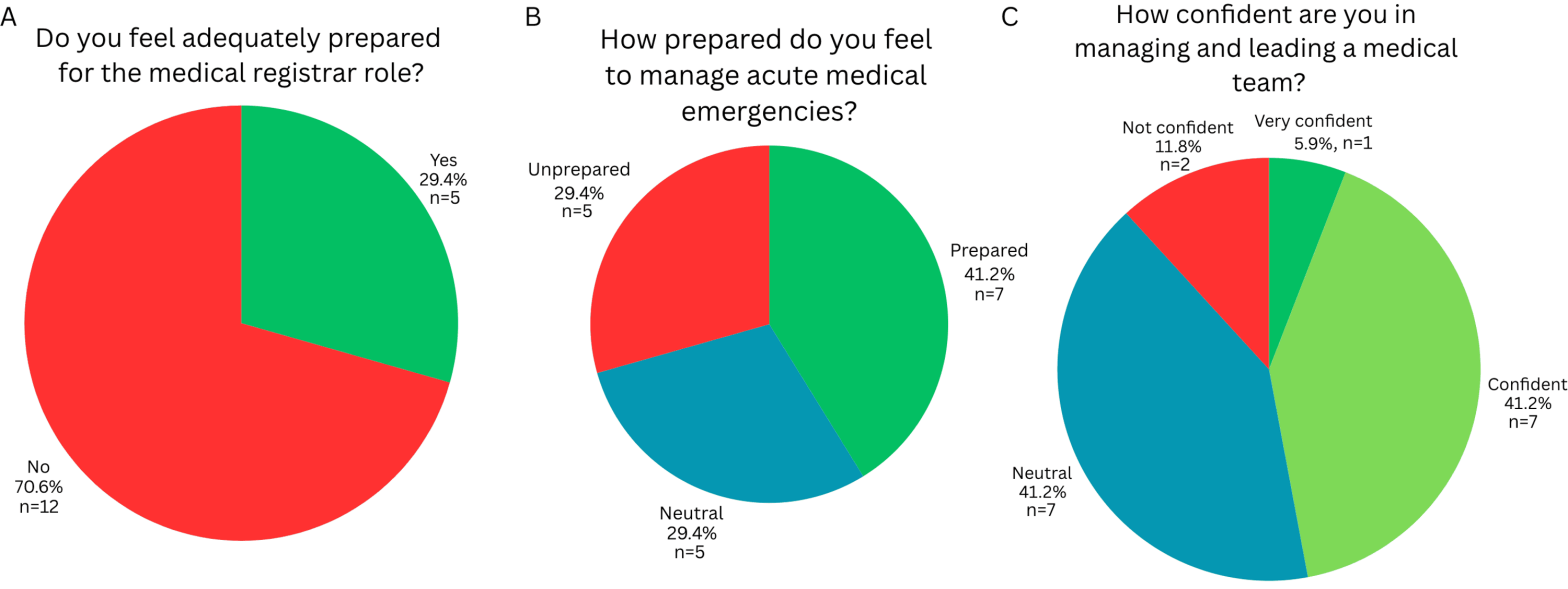
Perceived preparedness and leadership confidence among doctors working in or preparing for the medical registrar role (n = 17) (A) Adequate preparation for the registrar role; (B) preparedness to manage acute medical emergencies; (C) confidence in managing and leading a medical team

Seventeen doctors completed the needs assessment survey. The majority (70.6%, n = 13) of respondents felt inadequately prepared for the responsibilities associated with the medical registrar role (Figure 1A). An alarming figure given 47.1% (n = 8) of respondents had 1-3 years’ experience and 23.5% (n = 4) had over three years’ experience as a medical registrar.

The majority of participants felt prepared (41.2%, n = 7) or neutral (29.4%, n = 5) in handling medical emergencies or acute medical scenarios, with 29.4% (n = 5) reportedly feeling unprepared, as shown in Figure 1B. Despite 70.6% (n = 12) of respondents having more than one year working as a medical registrar, only 47.1% (n = 8) felt confident or very confident in managing and leading a medical team (Figure 1C). Only 58.8% (n = 10) had access to formal debriefing of difficult cases with senior registrars and consultants, but 88.2% (n = 15) believed regular debrief sessions would enhance their learning.

The majority (60%, n = 6) of SAS doctors and LEDs responding believed additional training on NHS systems would have eased their transition to registrar work. Notably, 9.1% (n = 1) of SAS and LED respondents had considered stepping down to an SHO role due to challenges experienced at the registrar level.

**Figure 2:**
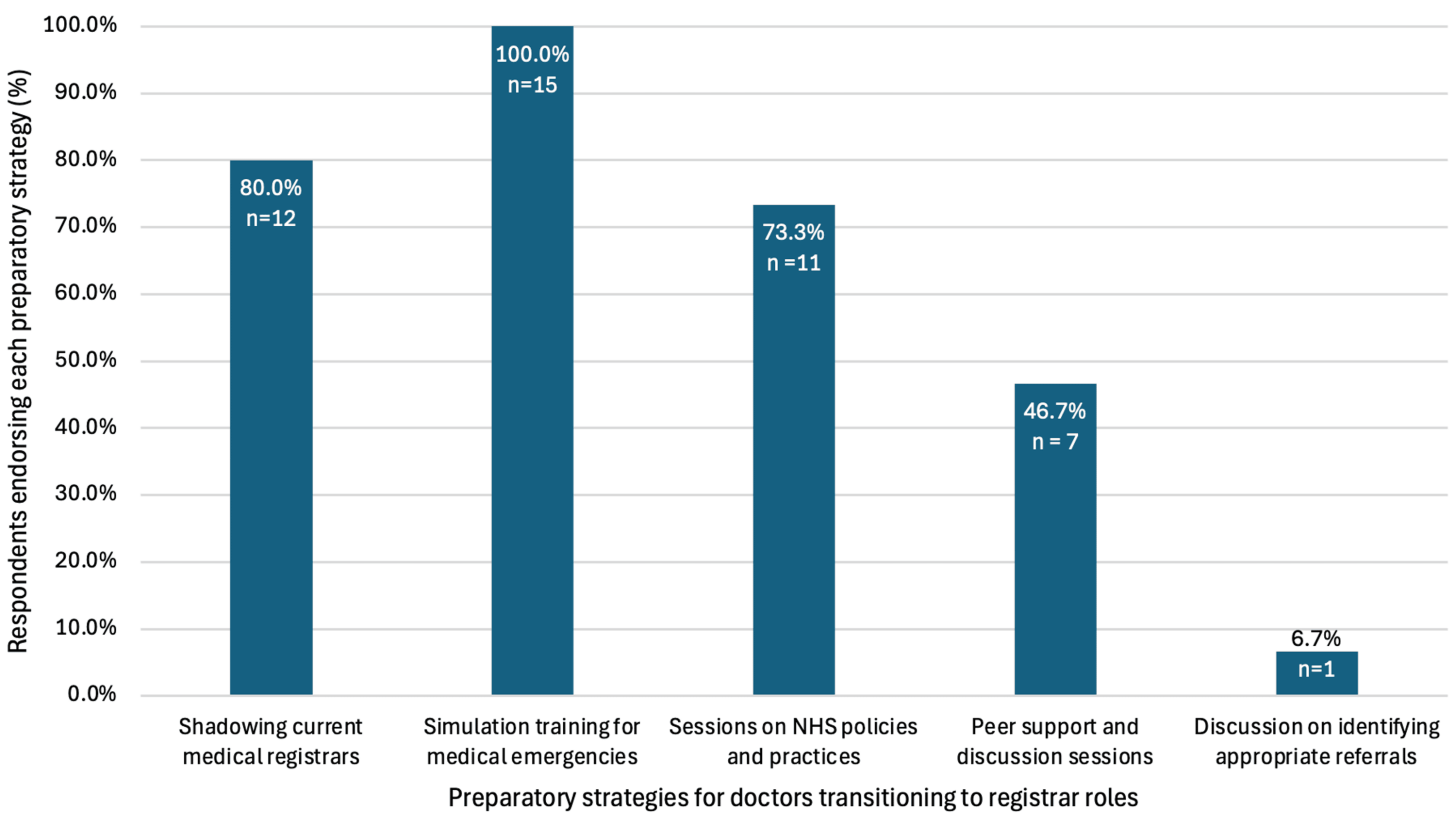
Preparatory strategies endorsed for doctors transitioning to registrar roles (n = 15). Simulation training for medical emergencies was most frequently identified (100.0%), followed by shadowing current registrars (80.0%) and sessions on NHS policies and practices (73.3%)

**Figure 3:**
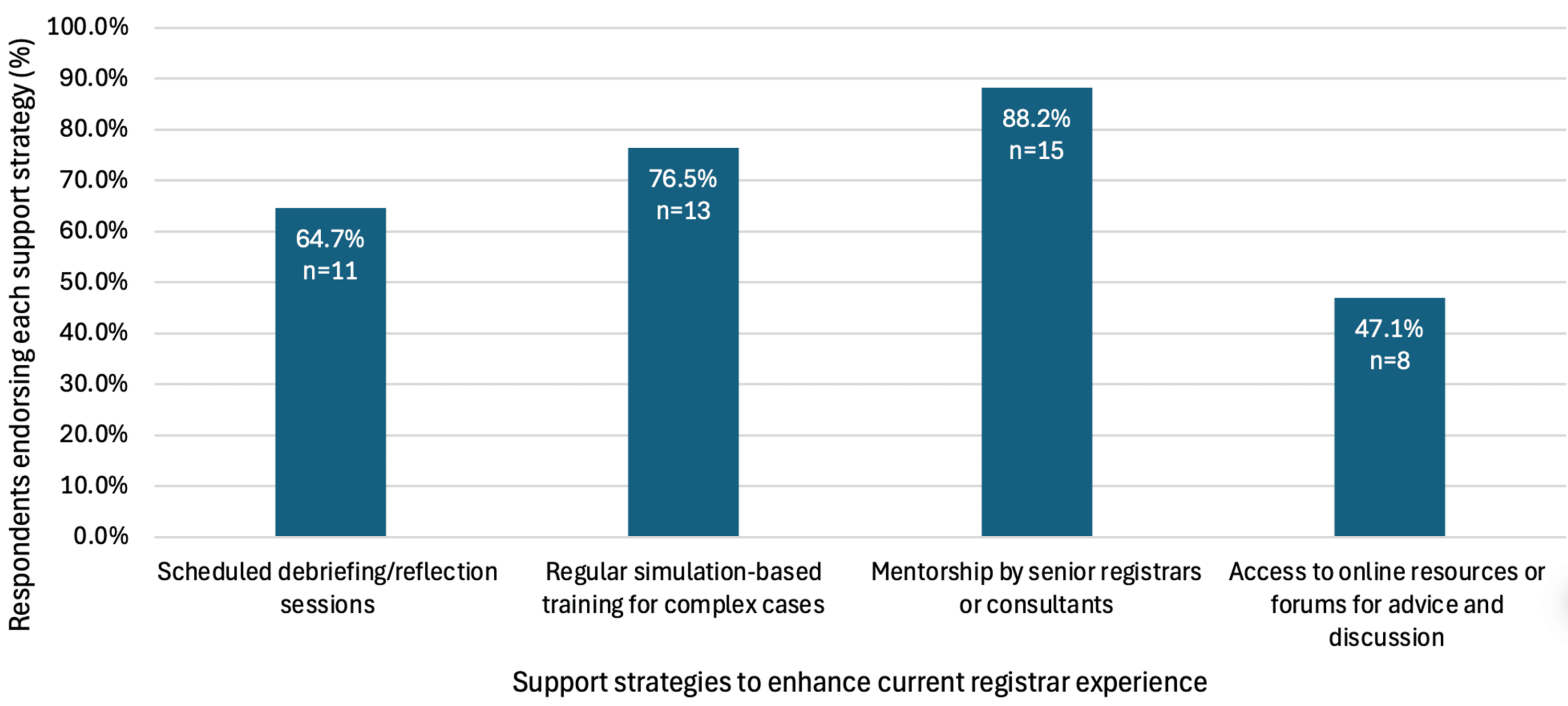
Support strategies endorsed to enhance the medical registrar experience (n = 17). Mentorship (88.2%) and simulation-based training (76.5%) were most frequently identified, followed by reflection sessions (64.7%) and online resources (47.1%)

All respondents (n = 15) indicated that prior to stepping up to the registrar role, simulation-based training would be beneficial, along with opportunities to shadow current registrars (80% (n = 12)) and sessions on NHS-specific policies and practices (73.3% (n = 11)) (Figure 2). Respondents indicated that the experience of working as a medical registrar can be improved through mentorship by senior registrars or consultants (88.2%, n = 15), regular simulation-based training for complex cases (76.5%, n = 13) and scheduled debriefing/reflection sessions (64.7%, n = 11) (Figure 3).

Step 2: immediate post-simulation feedback 

Six SAS medical registrars completed the immediate post-simulation evaluation. All participants rated the simulation scenarios as realistic and aligned with the demands of their clinical practice. Following the session, 100% (n = 6) reported improved confidence in managing acute presentations. Furthermore, 83.3% (n = 5) noted enhanced confidence in team communication and leadership under pressure. One participant highlighted that engaging in debrief and discussing different aspects of cases had made them think more broadly. All participants indicated they would recommend the session to colleagues and wished for similar future training. An unintended benefit of this simulation was the identification of a participant unable to perform to the level expected of a medical registrar, as recognised by senior medical staff, triggering targeted support.

Step 3: evaluation of impact in one-month follow-up

At one-month follow-up, four of the six simulation participants completed the self-evaluation survey. All respondents reported applying skills gained through the simulation in real clinical settings. Improvements were identified in confidence during on-call shifts, leadership in acute scenarios, task delegation, situational awareness, and communication. Participants also reported increased engagement in reflective practice, peer feedback, and self-evaluation. The primary barrier to applying these skills in practice was variability in team dynamics. Due to limited peer responses, it was not possible to reliably assess observed behavioural changes from colleagues' perspectives.

## Discussion

This study reports a lack of preparedness and confidence in the medical registrar role that may be addressed with interventions such as simulation, mentoring, and shadowing opportunities. High-fidelity simulation has been shown to be a valuable intervention in improving confidence and reflective practice in immediate and post-course evaluation. Furthermore, this intervention shows an approach to reduce disparities in access to postgraduate learning between trainees and non-training grade doctors, aligning with RCP recommendations for equitable educational opportunities between trainees and non-trainees [3].

The needs identified in the initial survey can be framed using Vygotsky’s Zone of Proximal Development [6]: enhanced supervision, mentoring, and shadowing enable consultants to provide the structure and feedback that supports registrars to practice safely at the limits of their current ability, thereby facilitating progression towards independent practice. This is particularly vital for the non-training grade cohort whose development can plateau without educational support. Medical registrars, in particular non-training grade registrars, rarely receive structured supervision or debriefing as highlighted in this study.

Although high-fidelity simulation is resource and time-intensive, protected sessions away from clinical demands and service pressures create a psychologically safe learning environment for participants and supervisors. This allows consultants to have dedicated time to observe and provide structured feedback on both clinical and non-technical competencies, opportunities rarely available in routine practice. Workplace-based learning encounters are more natural and more complex and tend to inspire independent learning [7]; however, workplace pressures limit supervisors’ ability to observe, provide feedback, and teach. Even if time permits, supervisor feedback through direct observation in the workplace imposes a high cognitive load on supervisors, who must simultaneously manage patient care and diagnostic tasks, exceeding working memory capacity according to cognitive load theory [8]. This divided attention can result in inattentional blindness and reduced focus on trainee performance [9], thereby diminishing the educational value of the encounter. Dedicated high-fidelity simulation sessions offer protected teaching time for supervisors to observe trainee practice, reducing cognitive load from the actual work environment, and guarantee focused assessment and meaningful constructive feedback through debriefing.

In addition to supporting focused feedback, simulation also serves as a valuable diagnostic tool for educators. In this study, the simulation environment enabled early identification of support needs and performance concerns as perceived by senior clinicians. Here, a participant’s difficulties were recognised through direct observation by senior clinicians in simulation, prompting targeted support, an outcome unlikely to emerge through conventional appraisal and existing performance evaluation tools. For SAS and LEDs, appraisals and 360° feedback can risk becoming procedural rather than developmental [10]; simulation offers a more meaningful, formative addition to performance evaluation. A combination of assessment tools, such as tests of medical knowledge, simulated encounters, and direct observation, was found to provide a more well-rounded evaluation of competence than single assessment tools [11]. Integrating simulation-based feedback could assist supervisors in the recognition of trainee needs, prompt early support and enhance the educational utility and validity of existing appraisal systems, particularly for those without structured training, such as LED and SAS cohorts.

One clear limitation of this study is the scale. It was conducted at a single district general hospital with a small cohort of SAS and locally employed medical registrars, which restricts the generalisability of the findings. Response bias may have influenced results, as those most engaged in professional development were more likely to participate. While performance bias in simulation must be acknowledged, a one-month follow-up indicated improved self-confidence and perceived behavioural change in clinical practice. Evaluation relied primarily on self-reported measures of confidence and preparedness rather than objective assessments of clinical or leadership performance. Although multi-source feedback was attempted, peer-feedback responses were limited, preventing a comprehensive Kirkpatrick level 3 evaluation of behaviour change [12]. Supervisor feedback was sought; however, as highlighted in the needs assessment, formal supervision is lacking in this cohort. Objective performance measures were not included, and future work could incorporate direct observational assessment or simulation-based performance metrics. Notably, while some participants reported feeling underprepared for the registrar role, none elected to step down to a more junior grade, highlighting the complexity of professional identity and role expectations within this cohort. Future iterations will aim to gather impact outcomes by informing participants that course organisers will contact participants’ educational supervisors for pre-course and post-course reports, and peer-feedback will be sought out by the course organisers rather than leaving the responsibility to participants. Despite these limitations, the study provides valuable preliminary insight into the educational needs of SAS doctors and supports the feasibility and perceived impact of simulation-based registrar training within similar hospital settings. Accordingly, conclusions should be interpreted as preliminary and reflective of perceived preparedness and confidence rather than objective measures of competence.

A qualitative study on medical registrars’ views on transitioning into their role as a transformative learning event highlights the lack of structured debriefing and guided reflection time with senior support [13], supporting the findings of this study. A further study showed that simulation-based training had a significant impact on acute medical registrars' clinical practice [14]. The study highlights that promotion of collaborative learning and self-reflection in a psychologically safe environment enhanced their educational experience and professional growth. Along with educational benefits, simulation-based education has a role in social cohesion and identity formation [15], which is particularly of importance to non-training grade SAS cohorts and those new to practising medicine in the UK. This study recommends high-fidelity simulation as a means of providing protected senior-led structured feedback, debriefing, and guided reflection time to support the development of confidence, reflective practice, and perceived preparedness. Furthermore, simulation-based training offers an opportunity for formative assessment to assist in support and appraisal, particularly for non-training grade doctors. Future work should explore longitudinal outcomes, integrating simulation-based formative feedback into formal appraisal processes to promote sustainable professional development among non-training registrars.

## Conclusions

This QI project demonstrates the feasibility of using high-fidelity simulation to support medical registrars, particularly non-training grade doctors, by enhancing perceived confidence and preparedness, identifying learning needs, and complementing existing appraisal processes. Within a small cohort, simulation provided a psychologically safe environment for structured debriefing, guided reflection, and meaningful senior observation. These elements are often constrained in conventional workplace-based assessments. The intervention enabled early recognition of support needs and facilitated targeted developmental measures that may not have emerged through conventional processes.

Given the small sample size and reliance on self-reported outcomes, findings should be interpreted as preliminary and reflective of perceived educational value rather than objective measures of competence. While further evaluation with larger cohorts and objective performance measures is required to determine the impact on clinical competence and patient outcomes, these findings suggest that simulation represents a feasible and potentially scalable educational approach. By utilising existing simulation infrastructure and providing protected teaching time, NHS Trusts may be able to better support SAS and LEDs in alignment with the Royal College of Physicians' recommendations, promoting more equitable educational opportunities and supporting safer patient care.
